# Case report: Acute pericarditis following hepatic microwave ablation for liver metastasis

**DOI:** 10.3389/fcvm.2023.1100916

**Published:** 2023-05-19

**Authors:** Anna Giulia Pavon, Vladimir Rubimbura, Anna Nowacka, Arnaud Hocquelet, Jurg Schwitter, David C. Rotzinger

**Affiliations:** ^1^ Centre of Cardiac Magnetic Resonance, Lausanne University Hospital and University of Lausanne, Lausanne, Switzerland; ^2^ Cardiology Division, Heart & Vessels Department, Lausanne University Hospital and University of Lausanne, Lausanne, Switzerland; ^3^ Cardio Surgery Department, Cardiothoracic and Vascular Division, Lausanne University Hospital and University of Lausanne, Lausanne, Switzerland; ^4^ Department of Diagnostic and Interventional Radiology, Lausanne University Hospital and University of Lausanne, Lausanne, Switzerland

**Keywords:** cardiovascular magnetic resonance, pericarditis, metastasis, microwave ablation (MW ablation), cardia tamponade

## Abstract

Hepatic microwave ablation (MWA) is a growing treatment modality in the field of primary and secondary liver cancer. One potential side effect is thermal damage to adjacent structures, including the pericardium if the hepatic lesion is located near the diaphragm. Hemorrhagic cardiac tamponade is known to be a rare but potentially life-threatening complication. Here we present the first case of cardiac complication following MWA treatment in a 55-year-old man who presented with late cardiac tamponade. Adequate and timely management is essential, and clinicians should be fully aware of the need to perform early transthoracic echocardiography to detect signs of pericardial effusion when cardiac involvement is suspected.

## Introduction

**Figure 1:**
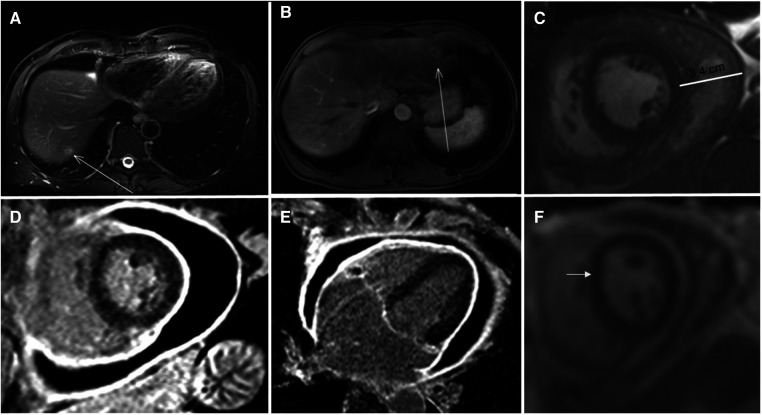
Panel **A**: MR of the abdomen showing a liver metastasis in the VII segment (arrowed) Panel **B**: MR of the abdomen showing a liver metastasis in the II segment (arrowed) Panel **C**: Steady-state free precession cine of a short axsis at mid-ventricular lever imaging showing the pericardial effusion Panel **D,E**: T1 weighted inversion recovery gradient echo image showing pericardial thickening (6 mm) and circonferential pericardial effusion. Panel **F**: Real time cine showing the initial shift of the interventricular septum (arrowed).

In the management of focal liver cancer, new approaches with reduced invasiveness have been developed, and image-guided thermal ablation is rapidly gaining acceptance in official therapeutic guidelines (1). Among them, microwave ablation (MWA) has been introduced, which is reported to be a safe procedure with no mortality and a low rate of major complications. On the other hand, the reported rate of cardiac complications with radiofrequency ablation (RFA) is 0.4% (2). Even though the needle temperature reached with MWA is higher than with RFA, no major cardiac complications following MWA have been reported so far, including in a large multicenter trial (3). We report the first case of cardiac complication following MWA, in the form of a late cardiac tamponade, which should raise the awareness regarding potential cardiac complications of MWA. Case Report: a 55-year-old man was admitted for hypotension and tachycardia to our emergency department. The patient had a history of colorectal adenocarcinoma with liver metastases in segment II (Figure 1, Panel A) and segment VII, treated with percutaneous MWA (Figure 1, Panel B) (1). The patient complained of short-term chest pain just after the MWA treatment, with spontaneous resolution. The ECG showed normal sinus rhythm and no signs of cardiac involvement were noted on clinical examination at discharge. A follow-up liver MRI obtained one month later showed a satisfactory treatment area and mild, asymptomatic pericardial effusion in a patient still asymptomatic for cardiac involvement. Thirty-four days later, the patient came to our attention because of progressive dyspnea and left shoulder pain. At physical examination, sinus tachycardia (HR 110 bpm) was present with a blood pressure of 100/60 mmHg. No signs of heart failure were present, and the rest of the physical examination was unremarkable. Blood test analysis showed a slightly elevated inflammatory marker (CRP 23 ng/L, upper limit 10 mg/L) and normal leucocyte count, compatible with the history of cancer. No sign of infection was present and renal function was normal (serum creatinine 87 umol/L). A chest x-ray showed signs of pleural effusion and an enlarged, globular shaped heart shadow suggestive of increasing pericardial effusion (Figure 1, Panel D). Transthoracic echocardiography confirmed the presence of a 6 cm circumferential pericardial effusion with compression of right chambers. Urgent pericardiocentesis was performed with resolution of symptoms, and 600 ml of serous fluid was drained; cytology showed no malignant cells. Due to the persistence of chest pain and dyspnea during the following days, a cardiac magnetic resonance (CMR) was performed. A relapse of pericardial effusion measuring 3.4 cm with the presence of fibrin was noted in steady-state free precession cine imaging (Figure 1, Panel E). On phase-sensitive late gadolinium enhancement (LGE) images acquired 10 min after intravenous injection of 0.2 mmol/kg of a gadolinium-chelate contrast medium (Gadobutrol, Gadovist, Bayer Healthcare, Berlin, Germany), diffuse and marked enhancement of the pericardium was found (Figure 1, Panel F and G). The pericardium was thickened (6 mm) with early signs of ventricular interdependence in real-time cine imaging (Figure 1, Panel H), consistent with a pattern of initial constriction. Due to the relapse of pericardial effusion, a collegial discussion determined that a pericardial window was necessary, which led to the complete drainage of the pericardial effusion. During the surgical intervention, another 500 ml of serous liquid was drained. The pericardium appeared visually thickened and fibrotic, in particular along the inferior wall of the left ventricle, close to the diaphragm. The patient was then treated with Non-Steroid Anti-Inflammatory Drugs (NSAIDs, ibuprofen 800 mg/day) and colchicine 1 mg/day for one month without recurrence. At 3 months follow-up a CMR was performed after NSAID withdrawal, and no signs of pericardial effusion or constrictive pericarditis were found.

## Discussion

Percutaneous RFA and MWA procedures have gained considerable importance in the field of liver tumor treatment, mainly due to their minimally invasive nature and the few risks associated with them (2). Cardiac complications following thermal ablation are rare but can lead to high morbidity and mortality. The low incidence of cardiac complications has been attributed to the highly perfused nature of myocardium, causing marked convective heat loss (heat sink effect), protecting tissue from thermal injury. Additionally, the continuous motion of the beating heart results in only intermittent and brief exposure to the heat, and the continuous movement of the diaphragm sliding over the fixed liver capsule could dissipate heat as well (4). Self-limited pericardial effusion may sometimes occur in clinical practice after MWA procedures, but no data in the literature determined its real incidence. As for now, only cases of hemorrhagic cardiac tamponade following RFA have been described, and these usually become apparent immediately after the procedure (3, 5). Theoretically, adjacent tissues may be damaged with MWA as well as with RFA, but despite the higher needle temperature used during MWA, no cases of cardiac complications have been reported so far, including in multicentric studies (4, 6). MWA has other advantages; to cover a large ablation zone, MWA requires the insertion of a single antenna, whereas two electrodes are needed to achieve the same coverage with RFA, which likely increases the risk of organ perforation and bleeding. Despite this, the case of life-threatening MWA complication we report calls for attention to care teams. Depending on the positioning of the MWA antenna, planning a follow-up visit to assess the presence of pericardial effusion might be appropriate. In summary, we presented a unique complication of late pericarditis complicated by cardiac tamponade following MWA treatment of a liver tumor in segment II. Thermal ablation procedures may damage adjacent tissue, and interventional radiologists should be aware of this complication even when using MWA instead of RFA. Since such side-effects can lead to life-threatening complications, careful follow-up of pericardial effusion is desirable. Prompt and appropriate medical treatment is essential and whenever cardiac involvement is suspected, clinicians should proceed to serial transthoracic echocardiography to rapidly detect signs of pericardial effusion and cardiac tamponade.

## Data Availability

The original contributions presented in the study are included in the article, further inquiries can be directed to the corresponding author/s.
